# The Putative Protein Methyltransferase LAE1 of *Trichoderma atroviride* Is a Key Regulator of Asexual Development and Mycoparasitism

**DOI:** 10.1371/journal.pone.0067144

**Published:** 2013-06-24

**Authors:** Razieh Karimi Aghcheh, Irina S. Druzhinina, Christian P. Kubicek

**Affiliations:** 1 Microbiology Group, Research Division Biotechnology and Microbiology, Institute of Chemical Engineering, Vienna University of Technology, Vienna, Austria; 2 Austrian Center of Industrial Biotechnology, c/o Institute of Chemical Engineering, Vienna University of Technology, Vienna, Austria; Universidade de Sao Paulo, Brazil

## Abstract

In Ascomycota the protein methyltransferase LaeA is a global regulator that affects the expression of secondary metabolite gene clusters, and controls sexual and asexual development. The common mycoparasitic fungus *Trichoderma atroviride* is one of the most widely studied agents of biological control of plant-pathogenic fungi that also serves as a model for the research on regulation of asexual sporulation (conidiation) by environmental stimuli such as light and/or mechanical injury. In order to learn the possible involvement of LAE1 in these two traits, we assessed the effect of deletion and overexpression of *lae1* gene on conidiation and mycoparasitic interaction. In the presence of light, conidiation was 50% decreased in a **Δ** *lae1* and 30–50% increased in *lae1*-overexpressing (OE*lae1*) strains. In darkness, **Δ** *lae1* strains did not sporulate, and the OE*lae1* strains produced as much spores as the parent strain. Loss-of-function of *lae1* also abolished sporulation triggered by mechanical injury of the mycelia. Deletion of *lae1* also increased the sensitivity of *T. atroviride* to oxidative stress, abolished its ability to defend against other fungi and led to a loss of mycoparasitic behaviour, whereas the *OElae1* strains displayed enhanced mycoparasitic vigor. The loss of mycoparasitic activity in the **Δ** *lae1* strain correlated with a significant underexpressionn of several genes normally upregulated during mycoparasitic interaction (proteases, GH16 ß-glucanases, polyketide synthases and small cystein-rich secreted proteins), which in turn was reflected in the partial reduction of formation of fungicidal water soluble metabolites and volatile compounds. Our study shows *T. atroviride* LAE1 is essential for asexual reproduction in the dark and for defense and parasitism on other fungi.

## Introduction

Comparison of the genomic inventory of *T. reesei*, *T. atroviride* and *T. virens* identified mycotrophy (i.e. successful feeding on either living or killed fungi) as the innate nature of the genus [1]. This lifestyle involves a combination of traits such as host recognition, attachment to and sometimes coiling around the host hyphae, and the secretion of antibiotic metabolites and cell-wall-degrading enzymes [2]–[4]. The molecular mechanisms involved have been studied mainly with regards to the possible involvement of hydrolytic enzymes (chitinases, glucanases and proteases) and secondary metabolites (gliotoxin, peptaibols, 6-pentyl-2H-pyran-2-one [6PP]) in antagonism, and of heterotrimeric G proteins and their receptors in sensing of host signals [5]. Mukherjee and Kenerley [6] reported the developmental regulator VEL1 (an orthologue of the *Aspergillus nidulans veA*, which encodes a conserved global regulator of development and secondary metabolism) [7], [8] regulates mycoparasitism that in *T. virens*.

In *A. nidulans*, most of the effects of VeA depend on the formation of a trimeric protein complex consisting of VeA, VelB and LaeA [9]. The latter protein, a putative *S*-adenosylmethionine-dependent methyltransferase, was originally described as a global regulator of secondary metabolism in several *Aspergillus* spp. [10], [11], and later on shown to be also required for the biosynthesis of secondary metabolites in the industrially applied fungus *Penicillium chrysogenum* (e.g. penicillin) and the phytopathogenic fungi *Fusarium fujikuroi, F. verticillioides* and *Cochliobolus heterostrophus*, respectively [12]–[15]. Further evidence emerged that LaeA also controls numerous developmental events in fungi, such as conidiation and fruiting body formation [12]–[14]. In plant and human pathogenic fungi, LaeA has also been demonstrated to be a virulence factor [13], [14], [16], [17].

We have recently studied the function of LAE1, the LaeA orthologue of *Trichoderma reesei* [18], [19]. Interestingly, in this fungus that has specialized to saprotrophic growth on pre-decayed wood, LAE1 is a major regulator for the expression of cellulases and hemicellulases that are required for feeding on this substrate [18], [19]. One may thus hypothesize that LAE1 controls different strategies to aid the fitness of the fungus in its environment.

As emphasized above, mycotrophy is the innate nature of *T. atroviride* [1]. In this work we have therefore tested the hypothesis that in *T. atroviride* LAE1 may be involved in mycoparasitic interaction of this species.

## Materials and Methods

### Fungal Strain and Culture Conditions

*Trichoderma atroviride* P1 (ATCC 74058) [20] was used throughout this work. For selected experiments, *T. atroviride* IMI 206040, and its *blr1* and *blr2* deletion mutants [21] were also used. It was grown on PDA (Difco™ potato-dextrose-agar) plates at 25°C. *Rhizoctonia solani* C.P.K. 3753, *Botrytis cinerea* C.P.K. 4679 and *Alternaria alternata* C.P.K 3737 were grown on 2% (w/v) potato dextrose agar (PDA) under 12 h cycles of light and darkness at 25 C.

*Escherichia coli* JM109 (Promega, Madison, Wisconsin) was used for plasmid construction and amplification.

### Manipulation of *lae1* Gene Expression in *T. atroviride*

To obtain mutants not expressing *lae1*, the 1.2 kb *lae1* coding region was replaced by the hygromycin B phosphotransferase (*hph*) gene from *E. coli* under *Trichoderma* 5′ and 3′ regularory signals [22]. To this end, 1.3 and 1.2 kb of the up- and downstream non-coding region of *lae1* were amplified using the primer pairs Patro_FW_ConMeth_ApaI (5′-TGGGCCCCATCATATCTGCTACTTGGCTC-3′)/Patro_Rev_ConMeth_XhoI (5′-TCTCGAGCGAGTATGGCGAGTCCTATAG-3′), and Tatro_FW_ConMeth_XhoI (5′-TCTCGAGGACCTAACCCGCATTACTTTG-3′)/Tatro_Rev_ConMeth_SmaI (5′- TCCCGGGCATCAAGAGCGTAGCACTG-3′) respectively. The two resulting PCR fragments were digested with ApaI/XhoI (upstream region) and SmaI/XhoI (downstream region), dephosphorylated and ligated into pBluescript SK(+) (Stratagene, La Jolla, California), previously cut with ApaI/SmaI, followed by the insertion of the 2.4 kb XhoI/SalI fragment of *hph* casette into the XhoI site resulting in pRKA_D 42103hph.

Vector pRKA_OE41617hph, which bears the *T. reesei lae1* gene under the constitutive expression signals of *tef1* [23] was used to generate *lae1* overexpressing strains of *T. atroviride*.

### Transformation of *Trichoderma*

Transformation has been carried out as described by Guangtao et al [24]. The strains were purified twice to obtain mitotic stability, and integration of the expression cassettes was verified by PCR analysis (**Fig. S1**).

### Assay for Growth and Conidiation

Cultures were grown on PDA at 25°C in a Sanyo incubator containing a Philips-master light source (TLD-15 W/840), either with illumination (12 hour cycles of light and dark; 1100 [±30] lux, 30 cm distance) or in full darkness (dark conditions), as specified. To this end, each plate was inoculated with a mycelial plug (5 mm diameter) taken from the edge of a 3-day-old non-sporulating plate. Three replica were done for each treatment. Conidia were harvested by gently rubbing them off in an equal volume of physiologically salt (0.1%, w/v, Tween and 0.8% w/v NaCl ), filtering through glass wool, and centrifugation (5000×g, 10 min). The conidia were then suspended in 2.5 g/l phytagel (Phytagel™^,^ SIGMA, Steinheim, Germany), mixed and their transmission measured at 590 nm in a Biolog standard turbidimeter. The number of conidia was calculated using a calibration curve with *T. reesei* conidia.

### Phenotype Microarrays

Growth of *T. atroviride* P1 and the *lae1* mutant strains derived from it on 95 carbon sources and water was investigated using Biolog® phenotype microarrays as described by Friedl *et al.* [25]. One-way or main-effect analyses of variance (ANOVAs) were used to compare the growth of selected strains on individual carbon sources. Tukey's honest significant difference (Tukey HSD) test as implemented in STATISTICA 6.1 was used for post hoc comparisons to detect the contribution of each variable to the main effect of the F test resulting from the ANOVA. Only p-values <0.05 were considered as significant.

### Assays for Fungal Antagonism

The ability of *T. atroviride* P1 and the respective recombinant mutants to antagonize and eventually parasitize other fungi was tested in dual confrontation assays on PDA plates as described earlier [26]. To study the ability to coil around hyphae of other fungi, 2% sucrose nutrient agar (SNA) (1.0 g/l KH_2_PO_4_, 1.0 g/l KNO_3_, 0.5 g/l MgSO_4_.7H_2_O, 0.5 g/l KCl, 0.2 g/l glucose, 0.2 g/l sucrose) was used, and the interaction monitored under the light microscope at the zone of interaction after 10, 14 and 21 days.

To test for a possible involvement of water soluble secreted compounds in antagonism, the *T. atroviride* strains were grown on PDA plates covered by cellophane. After the strains had covered approximately two thirds of the plates, the cellophane with *T. atroviride* was removed, and *A. alternata, R. solani* and *B. cinerea* were inoculated into the middle of these plates, and incubation continued until the respective controls reached 2 mm distance from the edge of the plates. The respective colony radius was monitored and compared to a control where the same fungus was pregrown.

### Triggering Conidiation by Mechanical Injury

To induce conidiation by mechanical injury [21], *T. atroviride* and its mutants were grown at 25°C on plates containing either minimal medium, Vogel’s medium or SNA in darkness. After the mycelium had spread over the whole plate, it was cut with a sterile cold scalpel and incubated in darkness for additional 24–48 hrs.

### Testing the Effect of Volatile Compounds (VOC)

*T. atroviride* P1 and its **Δ** *lae1* mutants were grown on PDA at 25°C for 72 h in periodic light and in darkness. Then the dishes were opened under sterile conditions and two dishes each arranged as an upside-down sandwich, in which the **Δ** *lae1* mutant plate was on the top. The sandwich was sealed with Parafilm tape and the incubation continued at 25°C for 8 days. Control plates (i.e. both plates contained the same strain) were always included.

The same test was applied when the effect of VOC on other fungi was tested. *A. alternata, R. solani* and *B. cinerea* were grown in the top plate.

### Hydrogen Peroxide Sensitivity

To test for sensitivity of the *Trichoderma* strains against hydrogen peroxide, they were grown on PDA plates supplemented with 0, 0.5, 1, 5 and 20 mM of H_2_O_2_, and incubated until the time that the fungi reached the edge of the plates. Changes in radial growth were measured 3 times per day.

### Assay for Cellulase Formation

The *Trichoderma* strains were grown in Mandels-Andreotti medium [27] containing 1% (w/v) carboxymethyl cellulose (CMC) as carbon source on 2% (w/v) agar plates. The inoculated plates were then incubated at 25°C for 3 days. Thereafter, they were incubated at 50°C for 18 hrs. The hydrolyzed cellulose was detected by staining with a 0.1% (w/v) Congo-Red solution, followed by subsequent washing with 1M NaCl. The hydrolysis of cellulose becomes visible by the bright halos around the fungal colony, and expressed as ratio of the diameter of the halo to that of the fungal colony. A ratio of 1 indicates absence of cellulase formation.

### Quantitative PCR

Following RNA isolation (using the RNeasy plant kit, Promega) 5 µg of the total RNA was treated with DNAse (DNase I, RNase free; Fermentas) and reverse transcribed (RevertAid™ First Strand cDNA Kit, Fermentas) using a 1∶1 mixture of oligo-dT and random hexamer primers. All quantitative RT-PCR experiments were performed on a Bio-Rad (Hercules, CA) iCycler IQ. For the reaction the IQ SYBR Green Supermix (Bio-Rad, Hercules, CA) was prepared for 25 ml assays with standard MgCl_2_ concentration (3 mM) and a final primer concentration of 100 nM each. All assays were carried out in 96-well plates. Determination of the PCR efficiency was performed using triplicate reactions from a dilution series of cDNA (1; 0.1; 0.01; 0.001). Amplification efficiency was then calculated from the given slopes in the IQ5 Optical system Software v2.0. Primers, amplification efficiency and T_m_ values are given in **Table S1**. Expression of the reference gene *tef1* was measured with both protocols for reference calculation. Expression ratios were calculated using REST© Software [28]. All samples were analyzed in at least two independent experiments with three replicates in each run.

## Results

### Identification of the LAE1 Orthologue of *T. atroviride*

We have recently identified LAE1 from *T. reesei*, and shown that it forms a supported clade with putative orthologues of *T. virens* and *T. atroviride* [18]. The annotation of the *T. atroviride* orthologue (Triat2∶302782; old number Triat1∶42103) was manually corrected (correct sequence deposited under NCBI GeneBank accession number KC174792). LAE1 is encoded by a 1328 nt ORF that is interrupted by 5 introns and encodes a putative 363 aa protein. The aa’s 110–205 specify the expected SAM-dependent methyltransferase domain (Pfam group PF13489).

### Phenotype of *T. atroviride* LAE1 Mutants

To investigate the function of *T. atroviride lae1*, we prepared knock out- and overexpressing strains (see Materials and Methods). Several mitotically stable **Δ** *lae1* and *OElae1* strains were obtained and verified by PCR (**Fig. S1**). Several **Δ** *lae1* strains and *OElae1* strains bearing one additional copy were then investigated with respect to growth and conidiation. Strains bearing the same mutation (**Δ** *lae1* or *OElae1* respectively; see below) displayed identical phenotypes. Despite several attempts we failed to introduce the wild-type *lae1* copy into the **Δ** *lae1* strains. Two **Δ** *lae1* or *OElae1* strains were thus selected for all further experiments, and gave essentially similar results (selected cases are shown in the supplementary information; see below). The **Δ** *lae1* strains showed a 25–30% reduced growth rate on plates with D-glucose or D-galactose as a carbon source, whereas growth of the *OElae1* strain was the same as that of the parent strain (data not shown). Otherwise the mutants did not show any morphological differences from the parent strain.

### LAE1 is Essential for Cellulase Formation in *T. atroviride*

In *T. reesei lae1* is essential for growth on cellulose and expression of cellulase and hemicellulase genes in *T. reesei* [18]. In order to learn whether this is also the case in *T. atroviride,* we grew the strains on plates with carboxymethyl cellulose as the only carbon source and analyzed cellulase secretion by Congo red staining. Indeed, *T. atroviride* P1 exhibited a ratio of halo diameter vs colony diameter of 1.29 (±0.011), which was slightly enhanced in the *OElae1* strain (1.47±0.036). The **Δ** *lae1* strain, however, yielded a value close to 1 (1.07±0.016) indicating no or only very little cellulase secretion (which was also reflected in the very small colonies). Thus the observed LAE1-dependent regulation of cellulase formation in *T. reesei* also extends to *T. atroviride*.

### LAE1 Effects Conidiation in *T. atroviride* in a Carbon Source and Light/darkness Dependent Manner

**Figure 1:**
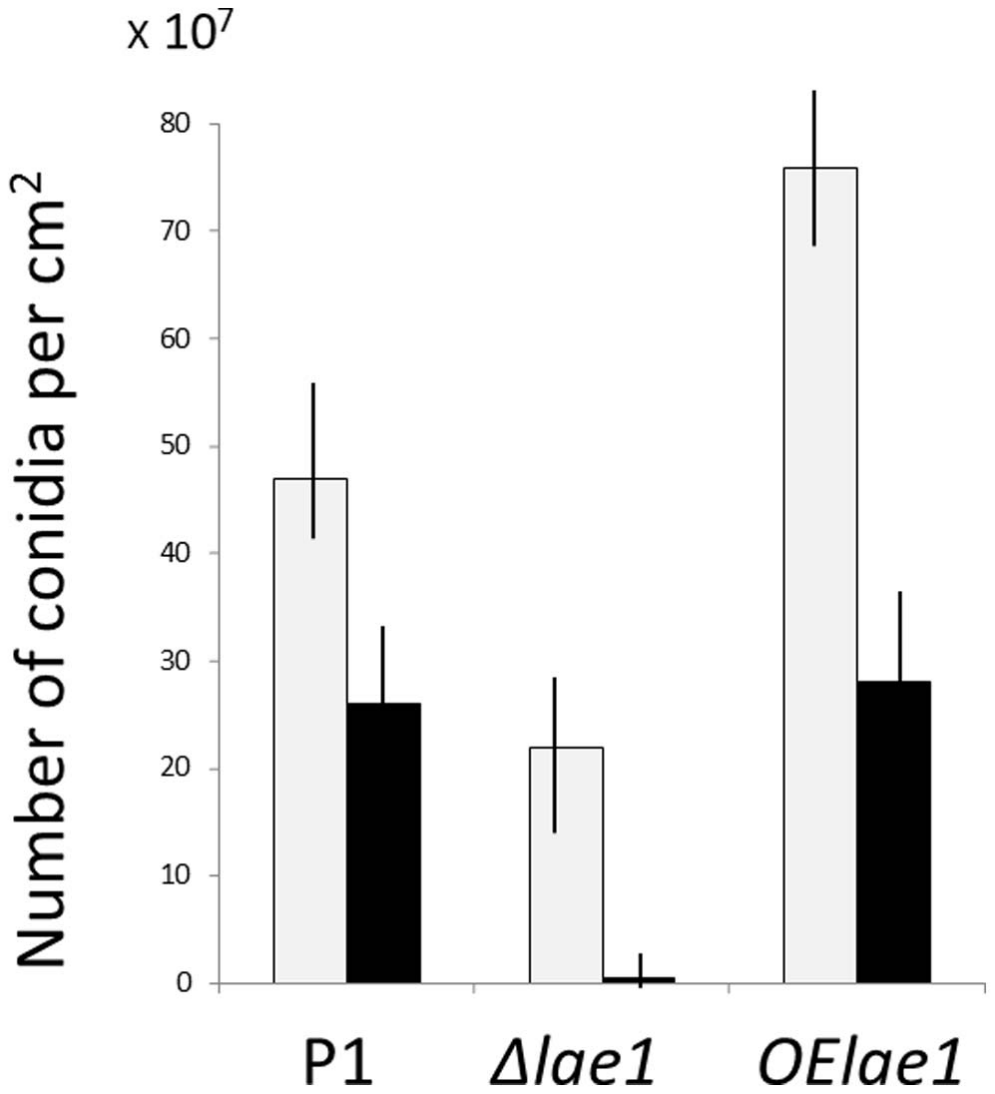
Asexual sporulation of *T. atroviride*. (A) Quantitation of conidiation of the parent (P1), **Δ** *lae1-1* and *OElae1* strains on PDA in light (white bars) and in darkness (full bars). Values are means of at least three independent biological experiments. Similar investigations with strain **Δ** *lae1-2* yielded values within ±8% of those of **Δ** *lae1-1.* All values are statistically different by the students t-test (p<0.05).

The loss-of-function of *lae1* of *T. atroviride* significantly affected the intensity of conidiation, albeit the effect differed in light and darkness (Figure 1): when cultivated on PDA in light, conidiation intensity was reduced by approximately 50% in the **Δ** *lae1* strain. Conidiation in the dark, however, was reduced to almost zero. The *OElae1* strains, on the other hand, exhibited an increased conidiation density under illumination, whereas it displayed the same level of conidiation as the parent strain P1 in darkness (Figure 1).

### Impairment of Conidiation in the *T. atroviride* **Δ** *lae1* Strain cannot be Rescued by Volatile Components from the Parent Strain

Conidiation in *Trichoderma* has been shown to be triggered by volatile compounds (VOC) from neighboring *Trichoderma* colonies [29]. We therefore surmised that the loss of conidiation in darkness could be due to a loss of the ability to form VOC. Consequently we tested whether VOC released by the parent strain of *T. atroviride* would rescue conidiation in the darkness in the **Δ** *lae1* mutant. However, this hypothesis had to be rejected: using an upside-down sandwich of two plates, in which the **Δ** *lae1* mutant was growing in the plate on the top and the parent strain P1 on the bottom, the **Δ** *lae1* strain maintained being unable to form conidia (shown for two mutants, **Δ** *lae1-1* and **Δ** *lae1-2* in **Fig. S2**).

### There is no Cross Talk between LAE1 and the Two Blue Light Receptors BLR-1 and BLR-2

**Figure 2:**
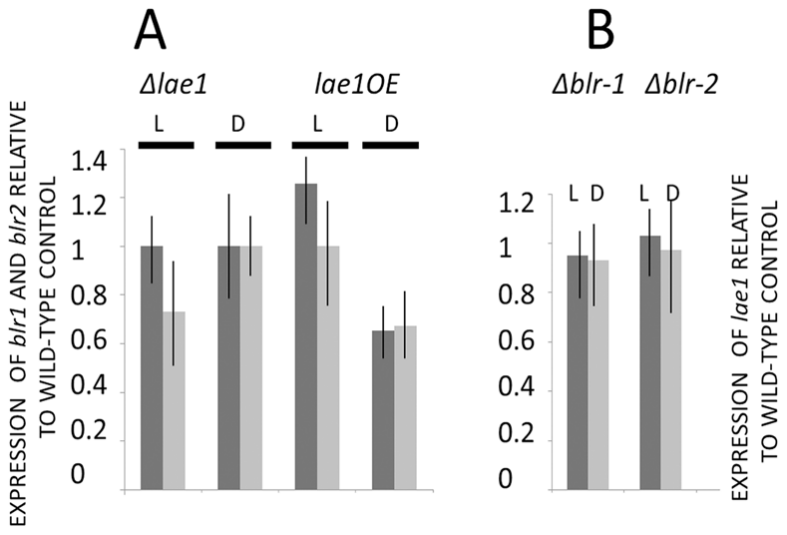
Effect of LAE1, and the blue light receptors BLR1 and BLR2 on each others expression: (A) Expression of *blr1* (dark grey) and *blr2* (light grey) in Δ*lae1-1* and *OElae1* in light (L) and darkness (D); (B) expression of *lae1* in Δ*blr1* and Δ*blr2* in light (L) and darkness (D). Vertical bars indicate the standard deviation (N ≥3). Expression of *lae1, blr1* and *blr2* was normalized to the expression of *tef1*. Relative gene expression is calculated as the ratio of the normalized expression in the mutant in –fold of that of the parent strain P1. None of the difference was found to be statistically relevant by students t-test (p>0.15).

The significant effect of the **Δ** *lae1* mutation on conidiation in response to light prompted us to investigate a possible cross-talk between LAE1 and the two blue light receptors BLR-1 and BLR-2, which form the top of the cascade that signals the presence of light to *T. atroviride* [21]. However, their transcripts were equally abundant in the parent, *OElae1* and **Δ** *lae1* strains (Figure 2 A) indicating that their expression is unaffected by *lae1* modulation. Also *lae1* was expressed at the same level in **Δ** *blr1* and **Δ** *blr2* mutants (Figure 2 B). Hence, *lae1* and *blr1/blr2* do not influence the expression of each other.

### LAE1 is also Required Triggering of Conidiation by Mechanical Injury

Conidiation in *Trichoderma* can also be induced by mechanical injury via generation of radical oxygen species (ROS) [21], [30]–[32]. We have therefore investigated if LAE1 is also required for conidiation triggered by mechanical injury in darkness. As shown in **Fig. S3,** mechanical injury resulted in conidiation only in the parent and *OElae1* strain but not in the **Δ** *lae1* strain, and LAE1 therefore influences sporulation also when triggered by mechanical injury.

### LAE1 is Required for Oxidative Stress Tolerance in *T. atroviride*

**Figure 3:**
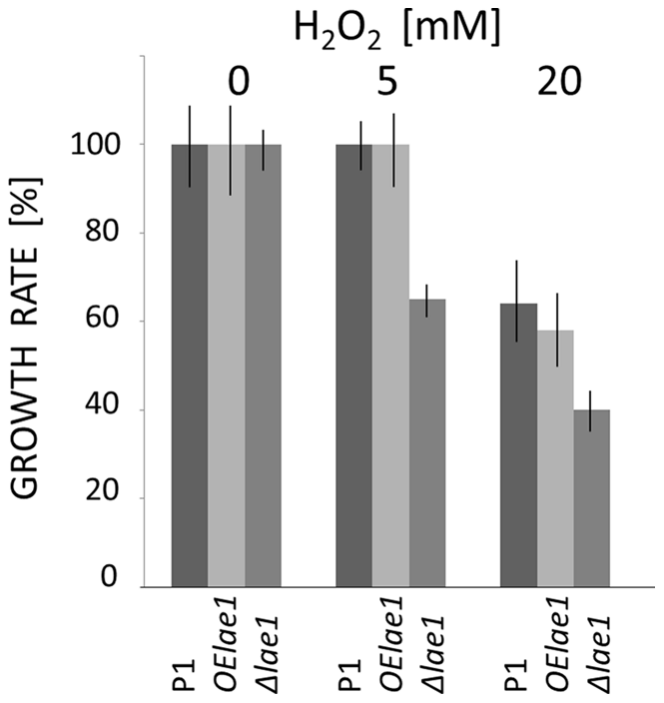
Effect of hydrogen peroxide on growth of *T. atroviride* parent strain and *lae1* mutant strains. Growth on PDA was monitored in intervals of 6–12 hrs for a period of up to 100 hrs. Growth rates were calculated from the phase where the increase in colony diameter vs time was linear and were calculated as mm/h. In the figure, the growth rate of the strain in the absence of hydrogen peroxide was set to 100%, and all other growth rates related to it. Data are means from at least 8 independent biological replicas.

Wu et al. [14] recently showed LAE1 is necessary for the oxidative stress response in the plant pathogen *Cochliobolus heterotrophus*. To find out whether LAE1 is required for the response to oxidative stress in *T. atroviride*, we tested the effect of hydrogen peroxide on the *T. atroviride* parent, the **Δ** *lae1* and the *OElae1* strain (Figure 3). The parent strain P1 proved to be resistant to hydrogen peroxide up to a concentration of at least 5 mM, and displayed about 60% of its original growth rate at 20 mM. Similar data were obtained for the *OElae1* mutant. The **Δ** *lae1* mutant, however, only showed 64% of its growth rate at 5 mM hydrogen peroxide, and exhibited only 39% of its original growth rate at 20 mM. Thus we conclude that LAE1 is partially involved in the defense against oxidative stress in *T. atroviride*.

### LAE1 is Essential for *T. atroviride* Antagonism and Defense against Other Fungi

**Figure 4:**
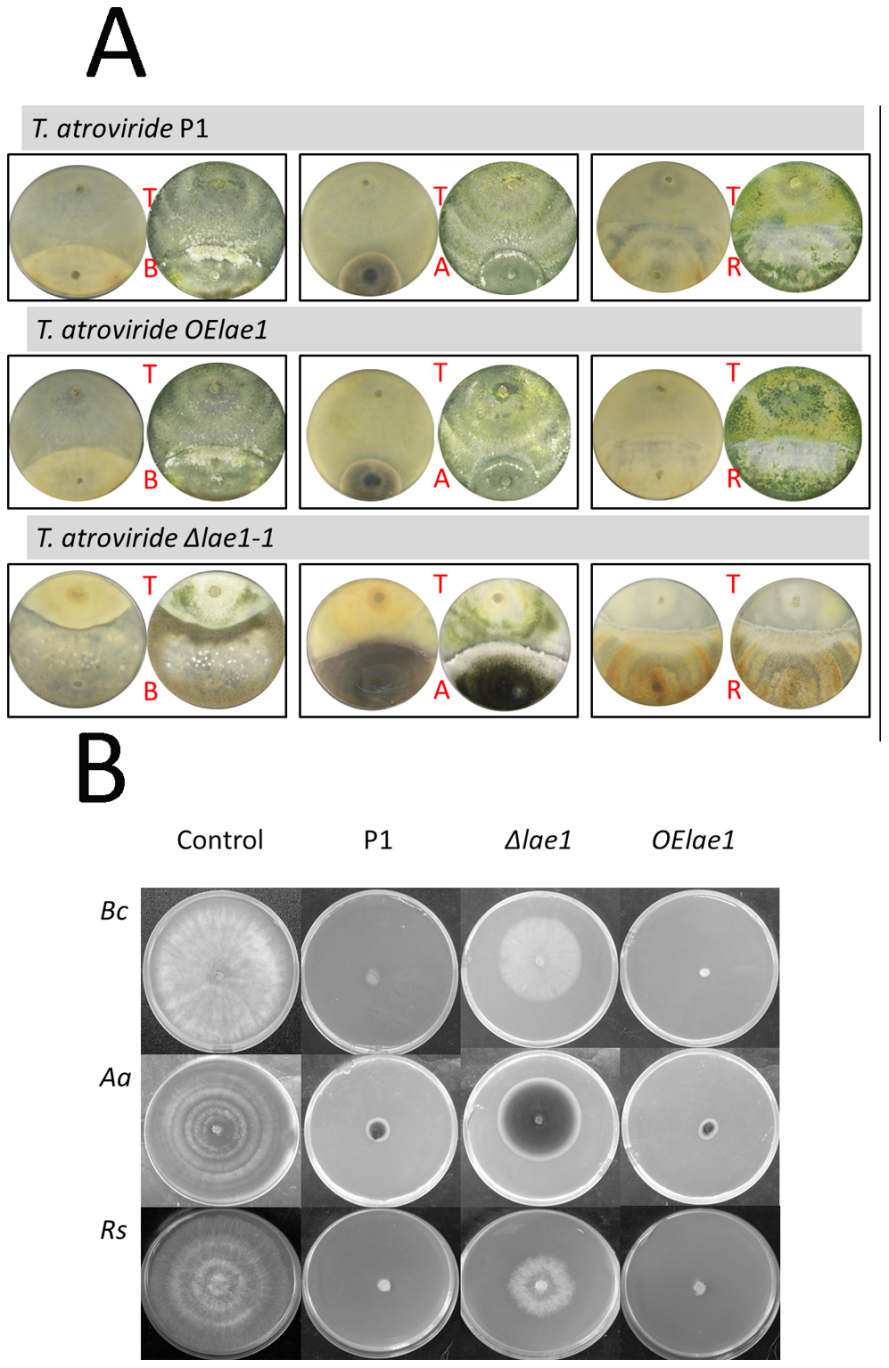
Phenotype of confrontation of *T. atroviride* P1 and the*lae1* mutants *OElae1* and Δ*lae1* (all T) against *B. cinerea* (B), *A. alternata* (A) and *R. solani* (R) after termination of growth of the latter three fungi. Left plates are photographed from the backside, right plates are photographed from top. (B) Test for production of WSC: *T. atroviride* parent strain, and the **Δ** *lae1-1* and *OElae1* mutants were grown on PDA agar covered by cellophane, and then removed and *Alternaria alternata* (*Aa*), *Rhizoctonia solani* (*Rs*) and *Sclerotinia sclerotiorum* (*Ss*) placed on these plates. The plates were photographed after 7 days.

To analyze whether LAE1 would be relevant for the mycoparasitic activities of *T. atroviride*, we confronted the parent strain P1, and the **Δ** *lae1* and *OElae1* mutants on plates with three standard model fungi used for antagonism experiments (i.e. *Alternaria alternata, Rhizoctonia solani*, and *Botrytis cinerea*). Their growth was monitored over the time in the presence and absence of *T. atroviride* and its *lae1* mutants (**Fig. S4 A**). As can be seen, all three test fungi were initially able to grow in the presence of *T. atroviride* and its *lae1* mutants at the same rate as in their absence, but stopped their growth when getting close (1–2 mm) to *T. atroviride* (plates for the second mutant strains shown in **Fig S4 B** ) This was about 50 h for all three fungi when confronted by strains P1 and *OElae1*, whereas it occurred in the **Δ** *lae1* mutant only after 65 hrs with *R. solani* and *B. cinerea* and 85 hrs with *A. alternata*. Correspondingly, the final colony diameter of these three fungi was higher when confronted with the **Δ** *lae1* mutant than with P1 or *OElae1*, which also corresponded with a smaller colony diameter of the **Δ** *lae1* mutant strain. However, in addition to this slower growth of the **Δ** *lae1* strain, visual examination of the plates (Figure 4; **Fig. S4B**) showed that it also failed to overgrow and feed on the tested plant pathogenic fungi, and in contrast its growth was suppressed by them. In confrontation with *R. solani*, *T. atroviride* **Δ** *lae1* almost completely also lost its ability to conidiate. In contrast, the mycoparasitic vigor of the *OElae1* strain was even increased, and we particularly noted an increased formation of coils around mycelia of *R. solani* (data not shown).

### LAE1 Regulates the Formation of Extracellular Antifungal Components

The dependence of mycoparasitism and antagonism on LAE1 prompted us to test whether this could be due to an involvement of LAE1 in the formation of water soluble extracellular compounds (WSC) that aid in the inhibition of growth of the plant pathogenic fungi. To this end, we grew *T. atroviride* P1, and its **Δ** *lae1* and OElae1 mutants on plates covered by cellophane. After *T. atroviride* had covered most of the plates, the fungal mycelium and the cellophane were removed, and *A. alternata, R. solani* and *B. cinerea* inoculated into the middle of these plates. As shown in Figure 4 B, the three fungi failed to grow on the plates on which the parent strain or the *OElae1* mutant had been pregrown, whereas they were still able to grow on plates on which the **Δ** *lae1* had been grown. Yet the latter growth was nevertheless clearly slower then on plates not precolonized by *Trichoderma*. Consequently we conclude that LAE1 contributes to but is not essential for the formation of extracellular antifungal compounds by *T. atroviride*.

### Loss of Function of *lae1* Decreases the Expression of Mycoparasitism-associated Genes

**Figure 5:**
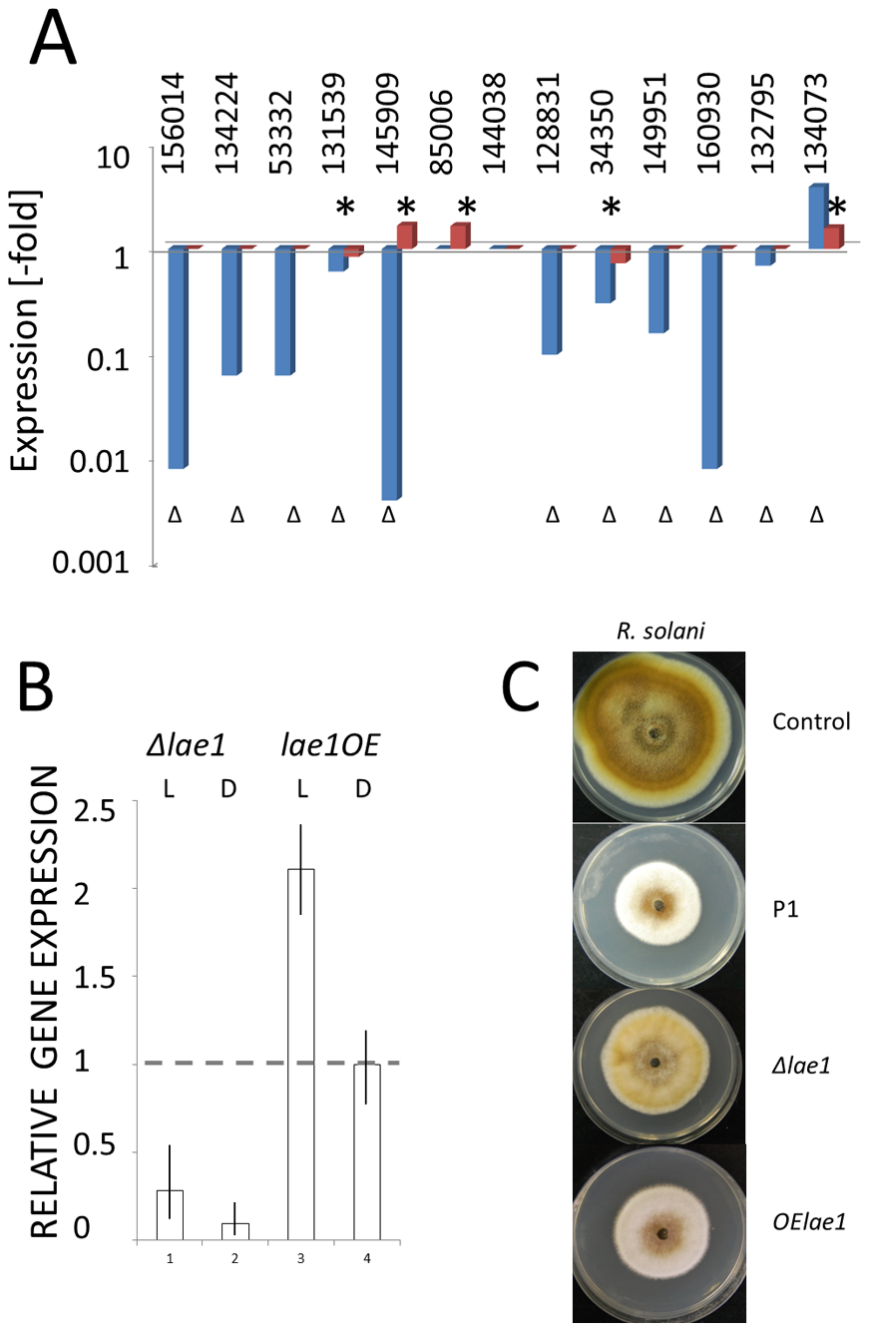
Modulation of expression of genes putatively involved in mycoparasitism (A). Ratios of expression between the parent strain and either the **Δ** *lae1-1* (blue bars) or the *OElae1* strain (red bars) are shown. The * and **Δ** symbols indicate p<0.05. Genes are given by their Triat2 number: 34350, GH16 ß-glycosidase; 144038, aspartyl protease; 160930, aspartyl protease; 128831, C-type lectin; 132795, C-type lectin; 134073, cyanovirin-N; 156014, GH16 ß-1,3/ß-1,4-glucanase; 85006, polyketide synthase (PKS); 53332, small cystein-rich secreted protein (SSCP); 131539, SSCP; 145909, subtilisin-like protease; 149951, subtilisin-like protease. Data are plotted relative to wild-type (P1) control. (B) Expression of the lipoxygenase gene in the **Δ** *lae1* and *OElae1* strains in light (L) and darkness (D). (C) Growth of *A. alternata* in the presence of VOC from the *T. atroviride* parent strain and the *lae1* mutants. Only *R. solani* is shown but essentially similar results were also obtained with *R. solani* and *B. cinerea.* Single plates from several (N>4) experiments are shown.

In order to learn whether loss of function of LAE1 would be due to a decreased expression of genes known to be associated with mycoparasitism, we have investigated the expression of 13 genes that were recently shown to be strongly upregulated during interaction of *T. atroviride* with *R. solani* [26]. These were two GH16 ß-1,3/1,4-glucanases, two aspartyl proteases, two subtilisin proteases, two polyketide synthases, two C-type lectins, one cyanovirin-type lectin and two small cysteine-rich secreted proteins. Figure 5 A shows that indeed 8 of these 13 genes were significantly underexpressed in the **Δ** *lae1* mutant. Interestingly, the expression of none of these genes was enhanced in the *OElae1* strain, implying that the superior mycoparasitic activity of this mutant (*vide supra*) cannot be due to the increased expression of any of these genes. Nevertheless, the data demonstrate that in *T. atroviride* LAE1 is necessary for the expression of some of the genes encoding extracellular hydrolases, secondary metabolites and proteins that putatively interact with other organisms.

### LAE1 Affects γ-pentyl-pyrone Formation by *T. atroviride*

One of the known antifungal metabolites produced by *T. atroviride* is 6PP [33], [34], which exhibits an intensive coconut smell. During the antagonism experiments, we observed this aroma in plates of the parent strain and even more in plates of the *OElae1* strain of *T. atroviride*, whereas it was absent from those of the two **Δ** *lae1* strains and only appeared faintly when this culture initiated its sporulation in the presence of light. To experimentally test whether LAE1 indeed regulates 6PP formation, we examined the expression of the lipoxygenase gene (Triat2∶33350) that is putatively involved in 6PP formation [3], and which is strongly upregulated during mycoparasitism [26]. In fact, the lipoxygenase transcript was strongly down regulated in **Δ** *lae1*, both in ambient light and in dark. It was also upregulated in *OElae1* in the dark but not in light (Figure 5 B).

To test whether reduced 6PP formation would contribute to the reduced antagonistic activity in the *T. atroviride* **Δ** *lae1* strain, we tested the effect of VOC from the parent strain, the *OElae1* and the **Δ** *lae1* strain on growth of the test fungi. The data show that VOC indeed reduce the growth of *R. solani* and also of *A. alternata* (data not shown), but only partially, and that this effect is much weaker in the **Δ** *lae1* strain (Figure 5 C).

## Discussion

The putative protein methyltransferase LaeA is still an enigmatic protein: it has originally been identified as a regulator of secondary metabolite (aflatoxin) biosynthesis [10], but subsequently was found to have – among other traits – also key functions in development: as an example, deletion of the *laeA* gene in *A. fumigatus* and *A. flavus* reduces conidiation [16], [35]. In *P. chrysogenum* and in *A. nidulans*, this impairment of asexual sporulation is seen both in light as well as in darkness [12], [19], whereas in *C. heterostrophus*, the *Chlae1* mutant was relieved from repression of conidiation in the dark and produced numbers of conidia similar to wild-type in light [14]. In *T. reesei*, whose sporulation is not enhanced by light [18], [36], formation of conidia is reduced to almost zero in both light and darkness. In the present study, we found that the *T. atroviride* **Δ** *lae1* mutant fail to conidiate in the dark on all carbon sources, while conidiation – which is stimulated by light [30], [31], [37] - was only reduced by 50% in light. Thus, while LaeA/LAE1 all affect asexual sporulation, the necessity for a functional LAE1 in *T. atroviride* prevails predominantly in darkness and appears to be partially counteracted by light, which is a unique case in the fungi studied so far.

One special trait of *Trichoderma* conidiation is that it can be induced by mechanical injury of the mycelium [21], [30]–[32]. The present results showed that injury-triggered sporulation of *T. atroviride* in the dark was also dependent on LAE1 function, as no conidia were formed in its absence. Mechanical injury has been shown to be triggered by an oxidative stress response caused by NADPH oxidase-dependent production of radical oxygen species (ROS), for which the proteins NOX1 and NOXR are essential [32]. We have not tested whether the expression of *nox1* and *noxR* is affected by LAE1 loss-of-function in *T. atroviride* (the *T. reesei* orthologues are not) [19] but tested whether – as in *C. heterostrophus* [14] – the **Δ** *lae1* strain is affected in its sensitivity to oxidative stress provoked by hydrogen peroxide. While we found that this indeed the case, the reduction of resistance against hydrogen peroxide was not complete and required much higher concentrations than in *C. heterostrophus* [14]. We therefore conclude that – even in the absence of *lae1* function - *T. atroviride* can still respond to oxidative stress. The complete loss of conidiation upon mechanical injury in the **Δ** *lae1* strain must therefore be due to a requirement for LAE1 by other components needed for this process.

One of them could be VOC formation: sporulation by *T. atroviride* has been shown to be triggered by VOC from other *Trichoderma* colonies [29], and Roze et al. [38] showed that in *A. parasiticus*, the Velvet A protein VeA is required for production of VOC that mediates asexual conidiation and sclerotia formation. Because of the known interaction of VeA with LaeA [8], [9], which has also been shown in *T. reesei* [19], we tested whether LAE1 would be involved in the stimulation of *T. atroviride* sporulation by VOC. However, VOC from the parent strain were unable to overcome the reduced conidiation in **Δ** *lae1*. Thus, either the effect of VeA on induction of sporulation is independent of LaeA (e.g. in *P. chrysogenum*, the VeA and LaeA orthologues PcVelA and PcLaeA have different and independent roles in asexual development) [12], or this process is differentially regulated in *A. parasiticus* and *T. atroviride*.

The most striking and not yet reported phenotype of loss-of-function of LaeA is that the *T. atroviride* **Δ** *lae1* strain had completely lost its mycoparasitic ability, and also the ability to defend itself against other fungi. This is similar to data that have been reported for **Δ** *vel1* strains of *T. virens*, suggesting that mycoparasitism is indeed controlled by the VEL1/LAE1/VEL2 (VeA/LaeA/VelB) complex [8], [9]. We should like to stress that *lae1* and *vel1* are so far the only genes that have been identified as global regulators of *Trichoderma* antagonism, and whose loss-of-function is not compromised by severe growth defects: deletion or silencing of other genes, such as those encoding G-proteins or their receptors, while also leading to impaired mycoparasitic activity, also caused significant reductions in the growth rate of *Trichoderma* [5] and it is therefore difficult to assess whether their effect on mycoparasitism is direct or indirect. Although the *T. atroviride* **Δ** *lae1* strains showed some reduction of growth on some carbon sources, the effects did not exceed ±30% of that of the parent strain under conditions of antagonism with the test fungi, and particularly the hyphal morphology was not significantly altered (data not shown). We therefore do not believe that the loss of antagonistic abilities could be solely due to this fact.

Interestingly, qPCR analyses showed that this loss of mycoparasitic ability correlated with a loss of expression of genes encoding cell wall hydrolases (GH16 glucanases), secondary metabolites (PKS), and proteins supposed to mediate hyphal contact to the host (lectins, SSCPRs). This would be in excellent agreement the current view of mechanism of *Trichoderma* mycoparasitism [1]. However, none of these genes displayed enhanced expression in the *OElae1* strain, and the superior mycoparasitic activity in *OElae1* strains thus remains unexplained. We have recently observed that overexpression of *lae1* even converts other *Trichoderma* spp. that exhibit only weak antagonistic activities, into vigorous mycoparasites (R.A. Karimi, M. Marzouk and I.S. Druzhinina, unpublished data). LAE1 therefore must act at a target that is central to the mycoparasitic response that still awaits identification.

Work on LaeA and its orthologues in several Aspergilli, but also in *P. chrysogenum, Fusarium fujikuroi, F. verticillioides* and *C. heterostrophus* has consistently proven that it regulates secondary metabolism [12]–[14], [16], [17], [35]. However, only a few genes of secondary metabolism were affected by a loss of function of *lae1* in *T. reesei* [19], suggesting that the function of LAE1 may have diverged in this genus. The present investigation with *T. atroviride* supports this view: while some of the secondary metabolism genes that have recently been shown to be upregulated during antagonism of *T. atroviride* against *R. solani* [26] were significantly underexpressed in the **Δ** *lae1* mutant, there was a less strong effect of LAE1 on the formation of inhibitory WSC and VOC, thus implying that the **Δ** *lae1* strain still can form WSC and VOC. Whether this is due to a decreased expression of several genes, or the complete blockage of expression of some of them remains to be determined. One must also bear in mind that the tests for WSC and VOC formation depends on the prior cultivation of *T. atroviride* in the absence of its prey, and it is possible that stronger effects may become apparent when the formation of these components is investigated under confrontation conditions. Yet it is clear from these studies that the lack of mycoparasitic activity in the **Δ** *lae1* strain cannot be explained by the observed changes in the expression of its secondary metabolism genes.

The present findings that LAE1 regulates the antagonistic and defensive reaction of the mycoparasite *T. atroviride*, is a further example of involvement of this protein in a specific response of a fungus to the environment. A similar conclusion has also been drawn by Sarikaya Bayram et al. [39], i.e. that LaeA is involved in the protective as well as the nutritional function for preparing the next generation for future life. Such a role would be in excellent agreement with an epigenetic function of LaeA/LAE1 [40], which however so far is only a speculation.

## Supporting Information

Figure S1 **Construction and proof for** ***T. atroviride OElae1*** **and Δ** ***lae1*** **strains: (A) constructs used to disrupt lae1 (top) and to express it under the tef1 promoter (bottom).** Numbers over the scheme indicate the size (in bp’s) of the promoter, ORF and terminator used; the number below the scheme of the nucleotide fragment amplified by the respective primers used. Bold numbers over the small bold arrows specify the primers used: 1, Patro_FW_ConMeth_ApaI; 2, Tatro_Rev_ConMeth_SmaI; 3, tef1SC; 4, TrLae1TermHind. For primer sequences see Materials and Methods of the main manuscript. (B) Identification of two **Δ** *lae1* strains among 8 transformants; the two arrows point to the 5 and 4 kb marker (M) band (from top); (C) Identification of OElae1 strains among 6 transformants and the P1 parent strain (track 7). The two arrows point to the 3 and 2.5 kb marker (M) band.
(TIF)

Figure S2 **Lack of induction of conidiation in** ***T. atroviride*** **Δ** ***lae1*** **by volatiles from strains P1,** ***OElae1*** **and Δ** ***lae1-1*** **and Δ** ***lae1-2*** **( = control) in the presence of light (L) or in darkness (D).**
(TIF)

Figure S3 **Triggering of conidiation in the** ***T. atroviride*** **parent and** ***lae1*** **mutant strains by mechanical injury.** The mycelium of the strains shown was cut with a scalpel and incubated under periodic illumination condition for 24 hrs. Single plates from several (N>4) experiments are shown.
(TIF)

Figure S4 **Effect of modulation of** ***lae1*** **expression on the ability of** ***T. atroviride*** **to inhibit growth of** ***R. solani, B. cinerea*** **and** ***A. alternata*** **.** A: (full ◊ indicate growth in the absence of *T. atroviride*; full **Δ** indicates growth in the presence of *T. atroviride* P1; full □ shows growth in the presence of *T. atroviride OElae1*; and × specifies growth in the presence of *T. atroviride* **Δ** *lae1*. Full arrows define the time point where *T. atroviride* P1 and *OElae1* stopped growth of the other fungi, whereas the dotted arrow specifies the time where *T. atroviride* **Δ** *lae1* strain stopped fungal growth. The solid and dotted horizontal line show the respective biomass formed by the three test fungi at the time of inhibition. B: confrontation of *T. atroviride* strain **Δ** *lae1-2* with *R. solani, B. cinerea* and *A. alternata.*
(TIF)

Table S1 **Primers used for qPCR analysis.**
(XLS)
