# Supplementary figures and images for: The Putative Protein Methyltransferase LAE1 of Trichoderma atroviride Is a Key Regulator of Asexual Development and Mycoparasitism
Source: PLoS One. 2013 Jun 24;8(6):e67144. doi: 10.1371/journal.pone.0067144 (PMC3691206; doi:10.1371/journal.pone.0067144)

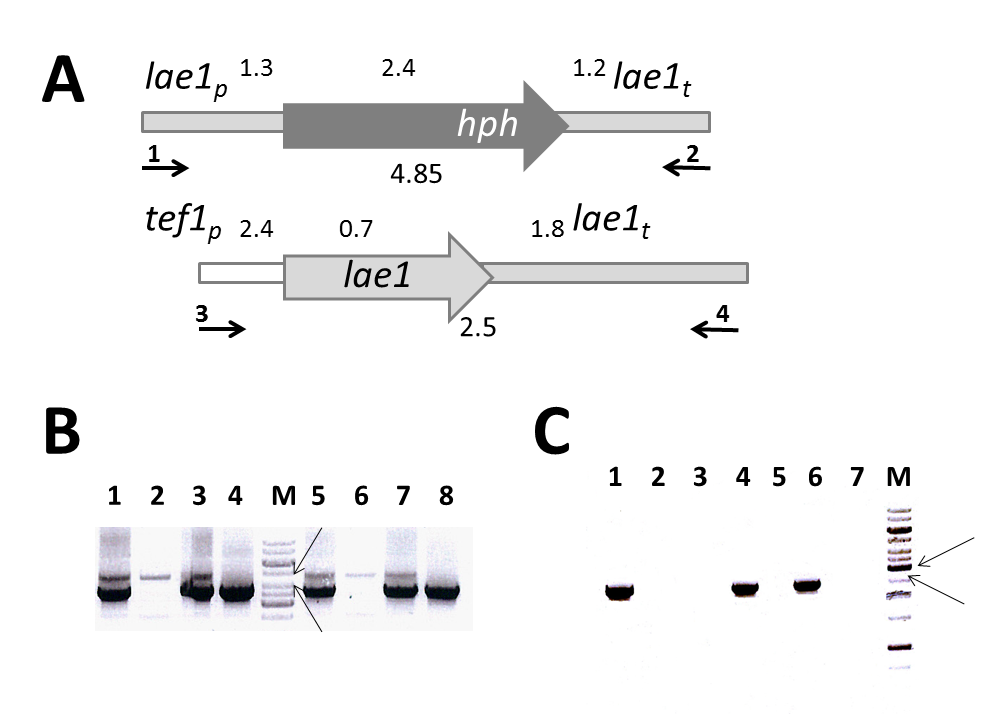

Supplement: Figure S1 — Construction and proof for T. atroviride OElae1 and Δ lae1 strains: (A) constructs used to disrupt lae1 (top) and to express it under the tef1 promoter (bottom). Numbers over the scheme indicate the size (in bp’s) of the promoter, ORF and terminator used; the number below the scheme of the nucleotide fragment amplified by the respective primers used. Bold numbers over the small bold arrows specify the primers used: 1, Patro_FW_ConMeth_ApaI; 2, Tatro_Rev_ConMeth_SmaI; 3, tef1SC; 4, TrLae1TermHind. For primer sequences see Materials and Methods of the main manuscript. (B) Identification of two Δ lae1 strains among 8 transformants; the two arrows point to the 5 and 4 kb marker (M) band (from top); (C) Identification of OElae1 strains among 6 transformants and the P1 parent strain (track 7). The two arrows point to the 3 and 2.5 kb marker (M) band. (TIF) [file pone.0067144.s001.tif]

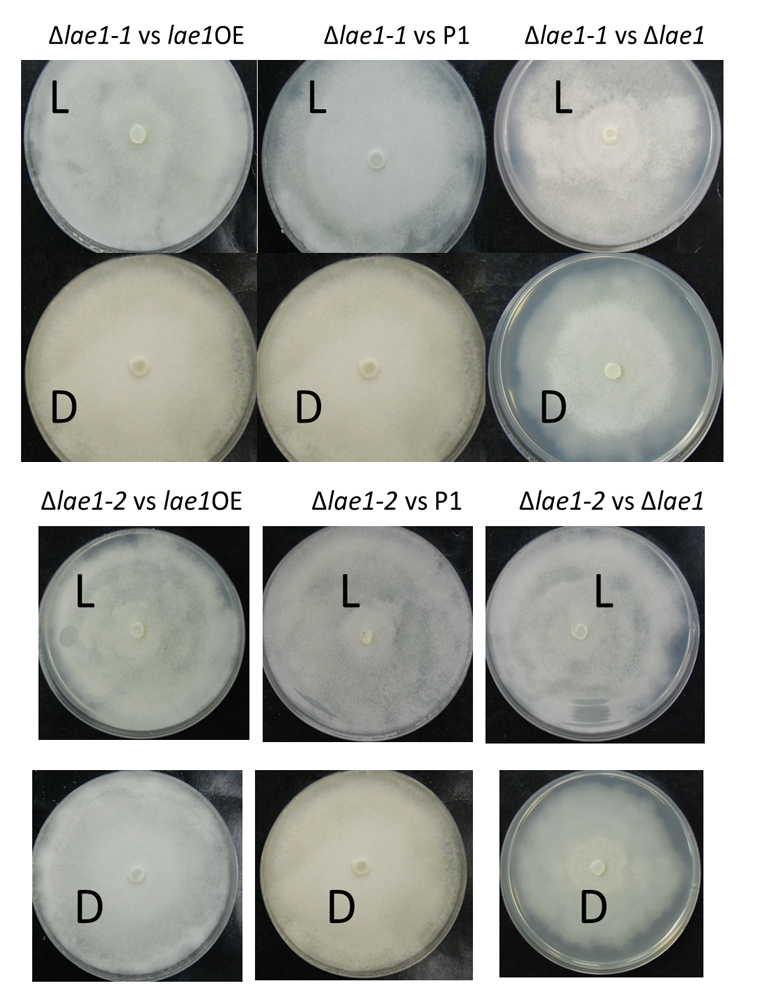

Supplement: Figure S2 — Lack of induction of conidiation in T. atroviride Δ lae1 by volatiles from strains P1, OElae1 and Δ lae1-1 and Δ lae1-2 ( = control) in the presence of light (L) or in darkness (D). (TIF) [file pone.0067144.s002.tif]

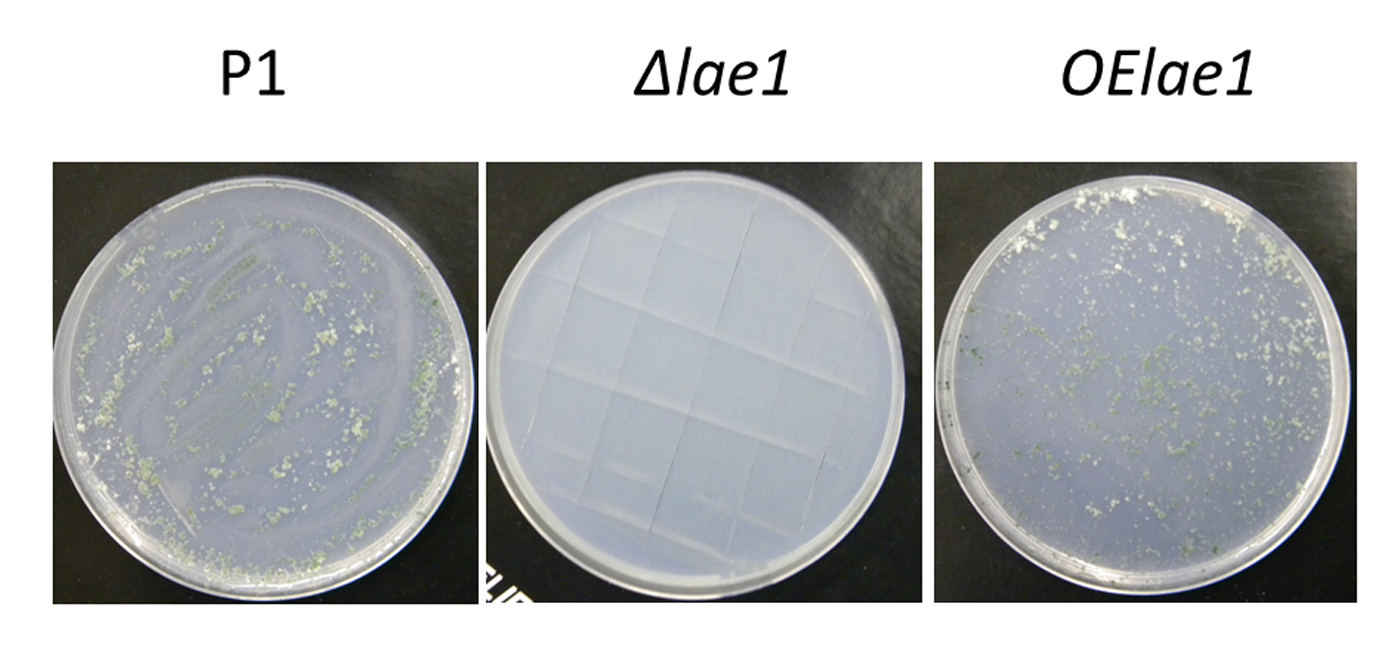

Supplement: Figure S3 — Triggering of conidiation in the T. atroviride parent and lae1 mutant strains by mechanical injury. The mycelium of the strains shown was cut with a scalpel and incubated under periodic illumination condition for 24 hrs. Single plates from several (N>4) experiments are shown. (TIF) [file pone.0067144.s003.tif]

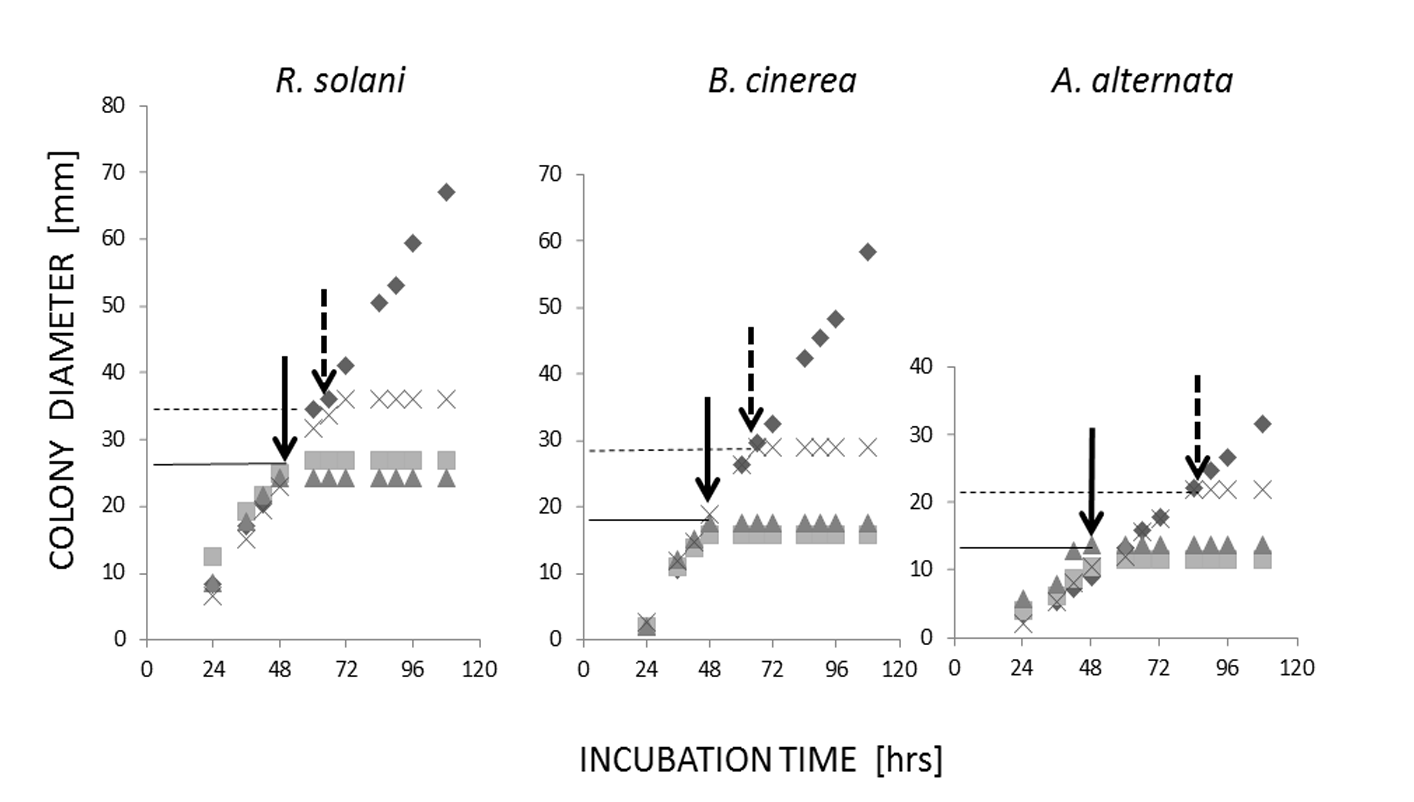

Supplement: Figure S4 — Effect of modulation of lae1 expression on the ability of T. atroviride to inhibit growth of R. solani, B. cinerea and A. alternata . A: (full ◊ indicate growth in the absence of T. atroviride; full Δ indicates growth in the presence of T. atroviride P1; full □ shows growth in the presence of T. atroviride OElae1; and × specifies growth in the presence of T. atroviride Δ lae1. Full arrows define the time point where T. atroviride P1 and OElae1 stopped growth of the other fungi, whereas the dotted arrow specifies the time where T. atroviride Δ lae1 strain stopped fungal growth. The solid and dotted horizontal line show the respective biomass formed by the three test fungi at the time of inhibition. B: confrontation of T. atroviride strain Δ lae1-2 with R. solani, B. cinerea and A. alternata. (TIF) [file pone.0067144.s004.tif]
